# Enhancing Bidirectional Encoder Representations From Transformers (BERT) With Frame Semantics to Extract Clinically Relevant Information From German Mammography Reports: Algorithm Development and Validation

**DOI:** 10.2196/68427

**Published:** 2025-04-25

**Authors:** Daniel Reichenpfader, Jonas Knupp, Sandro Urs von Däniken, Roberto Gaio, Fabio Dennstädt, Grazia Maria Cereghetti, André Sander, Hans Hiltbrunner, Knud Nairz, Kerstin Denecke

**Affiliations:** 1 Institute for Patient-Centered Digital Health School of Engineering and Computer Science Bern University of Applied Sciences Biel/Bienne Switzerland; 2 PhD School of Life Sciences Faculty of Medicine University of Geneva Geneva Switzerland; 3 ID SUISSE AG St. Gallen Switzerland; 4 Department of Diagnostic, Interventional, and Pediatric Radiology Bern University Hospital University of Bern Bern Switzerland; 5 Department of Radiation Oncology Bern University Hospital University of Bern Bern Switzerland; 6 ID BERLIN Berlin Germany

**Keywords:** radiology, information extraction, mammography, large language models, structured reporting, template filling, annotation, quality control, natural language processing

## Abstract

**Background:**

Structured reporting is essential for improving the clarity and accuracy of radiological information. Despite its benefits, the European Society of Radiology notes that it is not widely adopted. For example, while structured reporting frameworks such as the Breast Imaging Reporting and Data System provide standardized terminology and classification for mammography findings, radiology reports still mostly comprise free-text sections. This variability complicates the systematic extraction of key clinical data. Moreover, manual structuring of reports is time-consuming and prone to inconsistencies. Recent advancements in large language models have shown promise for clinical information extraction by enabling models to understand contextual nuances in medical text. However, challenges such as domain adaptation, privacy concerns, and generalizability remain. To address these limitations, frame semantics offers an approach to information extraction grounded in computational linguistics, allowing a structured representation of clinically relevant concepts.

**Objective:**

This study explores the combination of Bidirectional Encoder Representations from Transformers (BERT) architecture with the linguistic concept of frame semantics to extract and normalize information from free-text mammography reports.

**Methods:**

After creating an annotated corpus of 210 German reports for fine-tuning, we generate several BERT model variants by applying 3 pretraining strategies to hospital data. Afterward, a fact extraction pipeline is built, comprising an extractive question-answering model and a sequence labeling model. We quantitatively evaluate all model variants using common evaluation metrics (model perplexity, Stanford Question Answering Dataset 2.0 [SQuAD_v2], seqeval) and perform a qualitative clinician evaluation of the entire pipeline on a manually generated synthetic dataset of 21 reports, as well as a comparison with a generative approach following best practice prompting techniques using the open-source Llama 3.3 model (Meta).

**Results:**

Our system is capable of extracting 14 fact types and 40 entities from the clinical findings section of mammography reports. Further pretraining on hospital data reduced model perplexity, although it did not significantly impact the 2 downstream tasks. We achieved average *F*_1_-scores of 90.4% and 81% for question answering and sequence labeling, respectively (best pretraining strategy). Qualitative evaluation of the pipeline based on synthetic data shows an overall precision of 96.1% and 99.6% for facts and entities, respectively. In contrast, generative extraction shows an overall precision of 91.2% and 87.3% for facts and entities, respectively. Hallucinations and extraction inconsistencies were observed.

**Conclusions:**

This study demonstrates that frame semantics provides a robust and interpretable framework for automating structured reporting. By leveraging frame semantics, the approach enables customizable information extraction and supports generalization to diverse radiological domains and clinical contexts with additional annotation efforts. Furthermore, the BERT-based model architecture allows for efficient, on-premise deployment, ensuring data privacy. Future research should focus on validating the model’s generalizability across external datasets and different report types to ensure its broader applicability in clinical practice.

## Introduction

### Background

Radiology reports serve as a critical connector between medical imaging and patient care by condensing radiologists’ findings and interpretive insights into written text to inform physicians in charge. Historically, these reports have typically been narrative medical letters. While structured reporting has been shown to have several advantages and is therefore of increasing interest, most radiological reports are still presented in a narrative format [1]. This narrative style, while commonly rich in detail, often lacks the standardization seen in other areas of medical documentation. For example, laboratory reports are renowned for their ease of creation, enhanced standardization, and immediate comprehensibility [2,3].

Ideally, radiology reports would combine the depth and flexibility of narrative information with the clarity and structure of laboratory reports, allowing for a quickly comprehensible, easy, and unambiguous use for referring physicians. However, the style of radiology reports reflects the conflicting priorities of coping with high-throughput and standardized processes in radiology departments and providing individualized and patient-centered diagnostic information [4].

Structured reporting in radiology is defined as the systematic approach to creating reports using standardized language and formats, which can include templates, checklists, and hyperlinks [5]. However, the creation of templates for structured reporting is a labor-intensive process, and the supporting software platforms are struggling with accommodating the complexity and nuance of individual cases [6]. For referrers, the advantages of structured reporting are manifold, since the presentation of information in a structured format enhances clarity, reduces ambiguity, and thereby facilitates decision-making [4]. For radiologists, structured reporting can improve report quality, consistency, clarity, and completeness, potentially leading to a reduction in reporting errors and ultimately higher patient satisfaction [7].

One of the most prominent guidelines for standardizing reporting in radiology is the Breast Imaging Reporting and Data System (BI-RADS), developed by the American College of Radiology (ACR) [8]. BI-RADS provides a framework for describing mammographic findings, categorization of results, and recommendation of follow-up actions thereby aiding in decisions about management of patients with breast cancer [9]. The BI-RADS lexicon includes standardized descriptors for breast lesions, assessment categories that stratify the risk of malignancy, and management recommendations, all of which contribute to a more consistent approach to breast imaging interpretation and reporting [9]. Although BI-RADS provides a ruleset for reporting, mammography reports generally consist of semistructured sections of free text. Automatically extracting relevant information from these free-text sections to produce a structured report has the potential to improve reporting quality and eventually treatment outcomes. An opportunity to facilitate this process would be the application of large language models (LLMs), a deep learning–based model architecture trained on an extensive amount of text data, introduced in 2017 by Vaswani et al [10].

LLMs can be separated into encoder-based, sequence-to-sequence, and decoder-based models based on their architecture. While encoder-based models, for example, following the Bidirectional Encoder Representations from Transformers (BERT) architecture [11], excel in tasks requiring understanding and extracting information, decoder-based (or generative) models including the GPT model family [12], are designed to produce fluent, coherent text outputs. Sequence-to-sequence models, for example, the T5 (Text-to-Text Transfer Transformer) architecture, are best suited for tasks that involve generating new sentences based on a specific input [13]. LLMs continue to show impressive capabilities for realizing tasks related to natural language processing (NLP) in the medical domain [14,15]. They partly outperform clinicians in, for example, medical summary generation [16], answering clinical questions [17], and also the extraction of structured information from clinical text [18,19]. However, commercial state-of-the-art models continue to grow in size, requiring an increasing extent of scarce hardware resources and training data. Moreover, although large generative models currently show the best performance across many tasks and benchmarks, they are less explainable than, for example, encoder-based architectures [20]. Further limitations include models “hallucinating” wrong or misleading outputs [21] and restricted use of commercial models due to the sensitive nature of patient data [22]. Considering these limitations, the application of encoder-based LLMs for information extraction (IE) is of high interest for research in medical NLP due to their relatively small size and inherent output transparency. The possibility of reusing existing models and adapting them to the peculiarities of an institution—called further pretraining—additionally increases the potential of applying LLMs in radiology.

However, existing research on the specific task of information extraction from radiology reports based on LLMs is limited. Although during the last 5 years, several studies have been published, reporting quality and comparability of studies is impaired as shown by our previous work, a scoping review on studies describing information extraction from radiology reports [23]. It was shown that until August 2023, only pretransformer- and encoder-based models were applied. Moreover, LLMs might improve the generalizability of IE approaches. Common open challenges included missing validation on external data and augmentation of methodologies.

The authors of a recent systematic review on specifically BERT-based NLP applications in radiology concluded that the BERT architecture shows the “potential to elevate diagnostic accuracy, accelerate the generation of reports, and optimize patient care” [24].

Regarding the specific task of structuring mammography reports, Saha et al [25] conducted a scoping review on NLP applied to breast cancer reports: The authors show that NLP applications facilitate quality control in routine mammography and that LLMs offer distinct advantages due to their transfer learning capabilities and better performance. Before LLMs, rule-based approaches were most frequently used, as a systematic review on NLP-based extraction of cancer concepts from clinical notes showed [26].

Based on the existing literature, it becomes apparent that the number of extracted entities is usually limited and that the entity structure and types are proprietary for each IE project. To counteract these limitations, Steinkamp et al [27] introduced a method that enables clinicians themselves to define atomic, reusable, and standardized types of clinically relevant information called “facts” in 2019. A fact always corresponds to a continuous text span, comprising predefined entities, called “anchors” (unique keyword or phrase of a fact) and “modifiers” (optional, containing additional information). The authors based this information model on the linguistic concept of frame semantics, coined by Fillmore [28]. For implementation, they applied pretransformer, deep learning–based methods (bidirectional gated recurrent units). With this study, we refine this approach proposed by Steinkamp using a BERT-based architecture to improve model performance while keeping the scalability of the approach.

Frame semantics interprets words and phrases based on underlying conceptual structures, or “frames,” which provide essential context. Each frame represents a scenario with specific roles and relationships, showing how certain words trigger a network of related meanings [28]. Applied to information extraction in radiology, frame semantics enables the organized mapping of clinically relevant data by linking key entities. This structure helps to align extracted information with real-world clinical situations, making it possible to standardize complex medical findings and adapt them across various reporting formats and domains.

### Study Goals and Contribution

With this study, we want to investigate the application of encoder-based LLMs to build a pipeline that automatically extracts and normalizes clinically relevant information from mammography reports. We introduce a novel approach that integrates the linguistic concept of frame semantics with recent LLMs, enabling clinicians to define relevant information in a structured and reusable way. In addition, we study whether further pretraining can enhance model performance.

## Methods

### Overview

**Figure 1:**
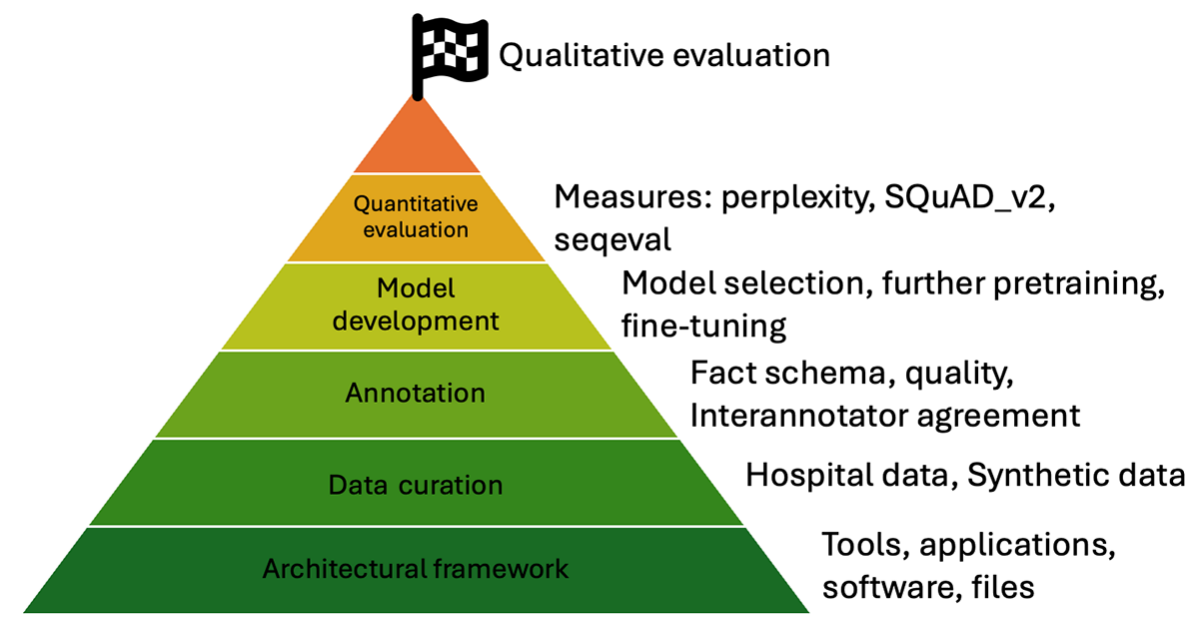
Methodology overview. SQuAD_v2: Stanford Question Answering Dataset 2.0.

This study implemented a previously introduced architectural framework for IE from clinical text [29]. In order to develop and evaluate our algorithms, our methodology further comprised the generation of real and synthetic datasets. Next, we designed and conducted an annotation subproject to obtain high-quality annotations of the documents according to a set of facts and associated modifiers. This annotated dataset then allowed us to develop different variants of models to automatically extract the specified information. Finally, we evaluated the models quantitatively, using standardized evaluation metrics, as well as qualitatively. To foster the traceability of our results, qualitative evaluation was performed by two radiologists using high-quality, clinician-written synthetic data, as the publication of the training dataset is prohibited due to institutional restrictions. We made the source code as well as the trained models and containerized pipeline image available (see data availability statement). Model pretraining and fine-tuning were performed on a single Apple M1 Max chip. We describe each step of our methodology in more detail below and provide an illustrated overview of the mentioned steps in Figure 1.

### System Architecture

In Table 1, we provide an overview of the applied technologies to implement the RadEx framework [29]. The framework describes the necessary components and artifacts and includes various specifications. These specifications include the underlying information model, the annotation process involving clinicians, the development of models to implement the IE tasks, and the use of the models in production [29].

**Table 1:** Implementation of selected elements of the RadEx framework for information extraction from clinical texts.

Framework component	Implementation
Fact schema definition user interface	Atlassian Confluence [30]
Integration server	Custom Java application
Template filling	ID MACS terminology server [31]
Inference server	BentoML [32]
Library	Python, Java, Github Packages [33]
Annotation generator	Custom Java application
Annotation tool	INCEpTION [34]
Report preprocessor	Custom Java application
Annotation analyzer	INCEpTION, Custom Java application
Type system converter	Custom Java application
Model repositories	HuggingFace transformers library [35]

### Dataset

We created separate datasets for self-supervised pretraining of LLM variants (“pretraining corpus”) and for subsequently fine-tuning these variants for the task of IE (“fine-tuning corpus”), see chapter “Model development.” Both datasets were preprocessed before application.

For the pretraining corpus, anonymized, raw hospital data provided as multiple .xlsx files were merged into a single .csv file and encoding errors (eg, German umlauts) were fixed using a custom Python script. Next, the file was symmetrically encrypted to allow pretraining on high-performance computing infrastructure outside the hospital.

The creation of the fine-tuning corpus of mammography reports also comprised the merging of raw data, stratification, and transforming data into the required format for annotation. The first step, data merging and cleaning, comprised merging raw data provided as multiple .xlsx files into a single .csv file, fixing encoding errors, removal of duplicates and nonmammography reports as well as tokenization and sentence splitting. Tokenization and sentence splitting were based on the open-source library, spaCy [36], augmented by a proprietary list of abbreviations and their associated expansions. Next, the reports were stratified according to the BI-RADS level of each report and split into 3 datasets for annotation guideline development, modeling, and validation. Each dataset contained an equal number of reports from each BI-RADS category, sampled by maximum variance within each category. Finally, each report was converted into the specified format (Common analysis structure [CAS]) according to the architectural framework.

For qualitative evaluation, 21 synthetic reports were created by a radiology resident and verified by a senior radiologist. This synthetic dataset comprised 3 reports per BI-RADS category (0-6). A sample of such a report is shown in Textbox 1. We used this dataset for qualitative evaluation and made it publicly available (see data availability statement; see Multimedia Appendix 1 for the original German version).

**Example of a translated synthetic mammography report with the Breast Imaging Reporting and Data System (BI-RADS) category 3.**

**Mammography in 2 views from October 11, 2017.**

Indication:

Positive family history (sister at 62 years). Palpable finding in the left breast.

Possible malignancy?

Findings:

No prior examinations.

Little fibroglandular tissue bilaterally.

Unremarkable skin and subcutaneous tissue bilaterally.

In the left breast, a circumscribed, round lesion, measuring 19×12 mm, located in the lower inner quadrant at 8 o’clock. No calcifications. Distance to the nipple is 54 mm. No other lesions in the left breast. No micro- or macrocalcifications.

Right breast without any lesions, micro- or macrocalcifications.

No suspicious lymph nodes.

Assessment:

Follow-up of the lesion on the left is recommended. Otherwise, no evidence of malignancy.

American College of Radiology Type B bilaterally

BI-RADS (Breast Imaging Reporting and Data System) 3 on the left

BI-RADS 1 on the right.

To assess how closely the synthetic reports resemble the real reports in terms of their semantic content, we adopted a cosine similarity-based methodology: We used gte-Qwen2-1.5B-instruct as an embedding model [37]. In detail, we sampled a stratified set of 21 real reports from the validation dataset. We then computed on the one hand the average similarity between this stratified set and the modeling dataset and on the other hand the average similarity between the 21 synthetic reports and the modeling dataset. These three datasets are equally distributed across the BI-RADS categories (0-6). To improve the comparability of this similarity-based approach, we additionally created a set of 21 German reports using ChatGPT (Free version used on March 9, 2024, with the prompt “Generate 21 radiology reports in German. They must be less than 700 words”) and also computed the average similarity between this LLM generated dataset and the modeling dataset.

### Annotation Process

The annotation process comprised 3 distinct phases: initialization, quality improvement, and final annotation. It was designed to maximize the quality of extracted information in terms of clinical relevance, and semantic and syntactic correctness. Below, we describe each phase in more detail.

The initialization phase was intended to define the use case–specific fact schema, describing the clinical information to be extracted from mammography reports, adhering to the underlying information model. Therefore, one computer scientist and one medical computer scientist (DR and JK) collaboratively created the first descriptive version of the fact schema based on the BI-RADS reporting guideline [8]. In addition, a first set of annotation rules, inspired by existing literature, was defined and documented [38]. Two clinicians (one radiologist, and one radiation oncologist) then iteratively augmented the fact schema with information not mentioned in BI-RADS. This fact schema was checked regularly for adherence to the underlying information model. The initialization phase resulted in an initial fact schema and corresponding annotation guideline, documented in a web-based, versioned collaboration platform (Confluence, Atlassian). For this phase, the annotation guideline development dataset was used.

The goal of the next phase, quality improvement, was to iteratively improve the fact schema and systematically revise the annotation guideline. Therefore, the coverage of the fact schema should be improved on the one hand, while on the other, interannotator agreement (IAA) should be maximized by augmenting and detailing the annotation guidelines. Quality improvement consisted of three iterations: In each iteration, 2 out of 4 clinicians applied the current fact schema and guideline version to annotate seven reports. The four clinicians included three physicians (two of them radiologists) and a medical student. After completion of the annotations, differences between clinicians were collaboratively discussed and resolved. Based on this discussion, the fact schema was adapted (addition, removal, or joining of facts, anchors, and modifiers) and the annotation guidelines were augmented, reducing ambiguities. In addition, IAA (Krippendorff α unitizing) was calculated on fact, anchor, and modifier levels for quantitative assessment. For this phase, the annotation guideline development dataset was used.

After the quality improvement phase, both fact schema and annotation guidelines were finalized and used to annotate the modeling dataset by a total of 3 clinicians. For each report in this dataset, only the clinical findings section was annotated. After completion, generated annotations were automatically checked for syntactic adherence to the fact schema. Errors were resolved manually by the respective clinician.

As an annotation tool, a local installation of the open-source annotation tool Inception was used [34]. During the project, the platform was migrated several times, spanning the versions 26.8 to 30. The tool provided all key functionalities necessary to conduct the complete annotation process. The tool-specific annotation configuration file was generated automatically based on the fact schema by a proprietary tool. Refer to Multimedia Appendix 2 for an example screenshot of the annotation interface.

### Model Development

As a basis for the fine-tuning process, we investigated 4 model variants, all based on the classical BERT architecture (110 million parameters). First, we applied medBERT.de, a BERT model further pretrained on ~4.7 million German medical documents, including medical texts, clinical notes, research papers, and other sources [39]. Second, we further pretrained medBERT.de by performing masked language modeling on the pretraining corpus. We refer to the resulting model as InselBERT_multi, as the pretraining corpus contained reports related to multiple modalities. “Insel” refers to the name of the university hospital from which the pretraining data was retrieved. Third, we further pretrained medBERT.de for 3 and 10 epochs on a subset of the pretraining corpus, containing only mammography reports. We refer to the resulting models as InselBERT_mammo_03 (pretrained for three epochs) and InselBERT_mammo_10 (pretrained for 10 epochs).

To extract clinical facts from mammography reports, a 2-step model pipeline was implemented to meet the specific needs of extracting information according to the fact schema: first, clinical facts (continuous spans of text) were extracted by fine-tuning the model variants for extractive question-answering (QA model); second, entities (anchors and modifiers) were extracted by fine-tuning model variants for named entity recognition (NER model), implemented as a sequence labeling task. For both models, the annotated modeling dataset was programmatically transformed into the required input format for each of the two tasks. We fine-tuned each of the two pipeline models using medBERT.de, InselBERT_multi, and both InselBERT_mammo variants.

### Model Deployment

After model development, the best-performing fine-tuned question answering (QA) and NER models (based on the averaged *F*_1_-score) were combined for inference using the open-source model serving framework BentoML (Atalaya Tech Inc) [32]. Using BentoML, model binaries, and associated files, serving logic and build configuration were packaged into a self-sustained unit (“Bento”), which was then containerized as a Docker image. This docker image was then deployed and exposed to an inference endpoint. This endpoint receives a mammography report and returns a structured JSON object containing the extracted facts and entities, including meta-information, for example, label probabilities.

To integrate the model output into a clinical context and encode it according to established medical terminologies, we used the commercial software ID MACS (ID Berlin) terminology server [31]. This approach enabled us to encode the extracted entities using Wingert and SNOMED-CT (Systematized Nomenclature of Medicine – Clinical Terms) terminologies.

To evaluate the encoding procedure, we developed a custom, Java-based application (Integration server) that sends a mammography report to the inference endpoint, receives the extracted facts and contained entities, and then sends each entity to the terminology server. The terminology server then returns the associated concepts from the specified terminologies.

### Model Evaluation

Our evaluation strategy comprises automated quantitative evaluation according to state-of-the-art evaluation metrics (pretrained model variants, fine-tuned QA model variants, and fine-tuned NER model variants) as well as a qualitative evaluation of the complete pipeline.

Further pretraining is evaluated by comparing model perplexity scores based on the validation split of the mammography corpus. Model perplexity is defined as “the level of perplexity when predicting the following symbol” [40] and computed as an exponent of the averaged loss obtained from model validation.

The QA model variants are evaluated on a held-out validation split (15%) by applying the SQuAD_v2 performance measure and bootstrapping [41]. According to the structure of the original SQuAD_v2-dataset, the training split only consists of examples with exactly one answer (fact instance) per question (fact type). Test- and validation set examples might have 1 answer per question. The sequence labeling model variants are evaluated on a held-out validation split (10%) by applying the seq_eval performance measure [42].

In addition to the automated and separate evaluation of each developed model, a qualitative evaluation of the complete pipeline is carried out based on the synthetic report set. For each report, all facts and entities are extracted in the first step. To furthermore investigate the potential of our pipeline to support standardization, each extracted entity is sent to ID MACS that returns concept candidates using 2 terminologies (SNOMED-CT and Wingert). All extracted entities and obtained terminology concepts are assessed by one radiation oncologist according to the following evaluation strategy: each extracted fact and entity was marked as correct or incorrect. Each obtained concept is marked as plausible or not plausible. Furthermore, manual error analysis is conducted.

Finally, we compare the qualitative evaluation results with those obtained using a generative approach, following state-of-the-art recommendations for prompt design. Specifically, we implement a three-shot prompting design, instructing the model to populate a report template based on the developed fact schema. The model is constricted to generate output in a specific JSON schema. We use Meta’s Llama 3.3 (70.6B parameters, Q4_K_M quantization) [43], the best-performing open-source model at the time of writing. The model is deployed on-premise on consumer-grade hardware (Mac Studio, Apple M2 Ultra, 24 cores, 192 GB memory), using Ollama as the inference platform [44]. One radiation oncologist then marks each extracted fact and entity as correct or incorrect.

### Ethical Considerations

The study, data acquisition, and use were approved by the local ethics committee (Ethics Committee Bern, BASEC 2022-01621).

## Results

### Overview

**Figure 2:**
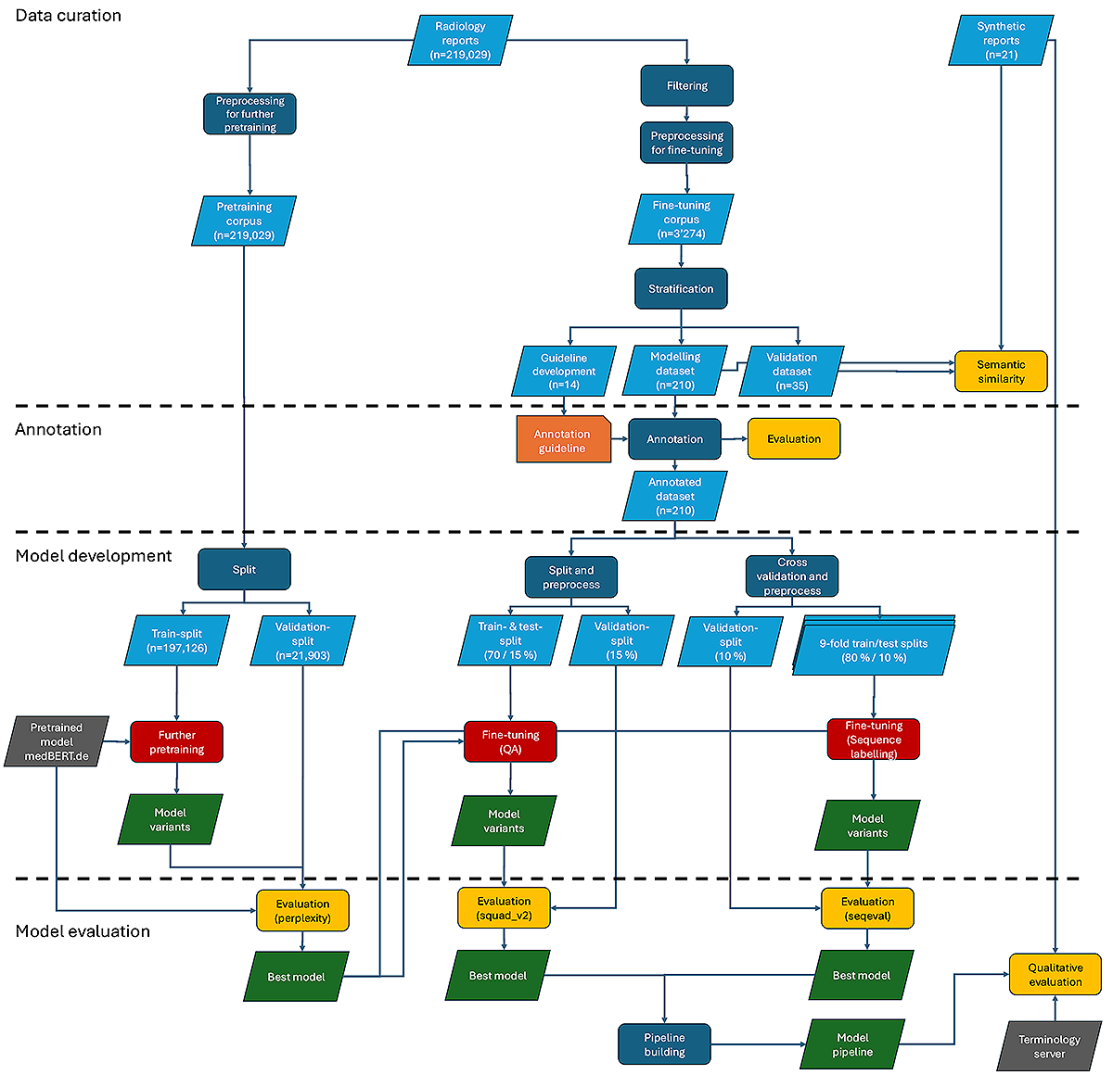
Overview of model development.

The previously described methodological steps can be grouped into 4 distinct phases: data curation, annotation, model development, and model evaluation (Figure 2). In the following subsections, we describe the results of the 4 phases.

### Data Curation

We used anonymized data covering the period of 2003-2023 provided by the University Hospital of Bern (Inselspital Bern). The pretraining corpus comprised 219,029 radiology reports, obtained from computed tomography, magnetic resonance imaging, x-ray, and sonography examinations. The mean number of tokens per report is 201 (SD 159), and the median is 156 (Multimedia Appendix 3). For further pretraining, the corpus was split into a training (197,126, 90% reports) and a validation (21,903, 10% reports) set.

The fine-tuning corpus consisted of 3274 mammography reports. The mean number of tokens per report was 158 (SD 63), and the median was 146 (Multimedia Appendix 4). Encoding errors were fixed in 217 reports, 389 duplicate reports, and 326 nonmammography reports were removed, resulting in a total of 2559 reports before stratification.

The final annotation guideline development, modeling, and validation dataset contained 14, 210, and 35 stratified reports, respectively. To allow batch inference of reports, a maximum document length of 512 tokens was defined, based on the fact that 99.5% of the report token length is 462 tokens, effectively ignoring eight reports during the sampling process.

A comparison of the synthetic dataset with the modeling dataset shows only a minor decrease in the cosine similarity score as compared to the validation set (76.9% and 79.1%, respectively). In contrast, the comparison of the ChatGPT-generated reports with the modeling dataset show a clear reduction of the cosine similarity score (57.3%).

### Annotation

The IAA results of the three rounds during the quality improvement phase are shown in Table 2. A total of 210 mammography reports (the modeling dataset) were annotated according to the finalized fact schema and annotation guideline. For each report, only the clinical findings section was annotated. Therefore, out of 24 facts and 66 modifiers defined in the schema covering a complete mammography report, 4 facts (+corresponding anchor) and 26 modifiers were not annotated, which is not present in the 210 annotated reports. A total of 6 facts (+corresponding anchor) and 14 modifiers that were annotated less than twenty times (defined as the minimum class frequency), were additionally excluded from the data. The annotated modeling dataset comprised 14 fact types and 40 entity types (26 modifier types and 14 anchor types) for the domain of mammography, resulting in a total of 2519 annotated fact instances (Table S1 in Multimedia Appendix 5) for details regarding the frequencies of annotated facts and modifiers as well as their original German definitions.

**Table 2:** IAA^a^ (Krippendorff’s alpha unitizing) after each annotation iteration of 7 new reports.

Annotation round	IAA facts	IAA anchors	Fact (=anchor) classes annotated (n/n)	IAA modifiers	Modifier classes annotated, n
Round 1	0.49	0.42	24/—^b^	0.50	46
Round 2	0.74	0.64	22/21	0.60	45
Round 3	0.83	0.61	23/22	0.61	48

^a^IAA: interannotator agreement.

^b^Not applicable.

### Model Development

Masked language modeling was performed for 3 epochs, applying a learning rate of 2**×**10^–^⁵, a weight decay of 0.01, and a masking probability of 0.15.

For implementing the QA model, the annotated modeling dataset was further separated into a train, test, and validation split, containing 70%, 15%, and 15% of reports, respectively. Next, data were transformed into the squad_v2 format. This in turn resulted in 1972, 274, and 273 examples per set, respectively. While the train and test set entries each contain only one question (fact type) with its associated answer in the text (fact instance), the validation set entries might contain 1 associated answer. This is always the case when one report contains multiple fact instances from the same fact type. For a split generation, shuffling and seeding were applied.

Fine-tuning of the QA model was implemented based on an existing repository for extractive question answering [45]. Hyperparameters included a learning rate of 3**×**10^–5^, 5 training epochs, a maximum sequence length of 384, and a document stride of 128.

For the NER model, each annotated fact was transformed into an annotated training example, where each token of the fact was annotated according to the IOB format [46]. The 210 annotated reports contained 2772 facts. 9-fold cross-validation was applied for model training, each fold comprising 80% train data, and 10% test data. The same validation split of 10% of the data was shared among folds.

The PyTorch-based implementation of the NER model is based on the HuggingFace Transformers and Trainer application programming interface. Hyperparameters were defined as follows; a learning rate of 5**×**10^–5^, a maximum of 100 training epochs, a weight decay of 1**×**10^–2^, a batch size of 16, and the default AdamW optimizer. A seed was set manually. An early stopping strategy aborted the training process if model performance as measured on the test set dropped during 5 consequent epochs—in that case, the best model was saved, loaded, and final evaluation was performed on the held-out validation set.

### Quantitative Model Evaluation

Further pretraining of model variants resulted in a reduction of the model perplexity score as shown in Table 3, obtained on an independent test set corresponding to 10% of the available training data.

Table 4 shows the results of fine-tuning each of the three further pretrained model variants for extractive question answering including confidence intervals based on bootstrapping (599 samples), compared to the baseline model (medBERT.de). Table 5 shows the corresponding results of fine-tuning for sequence labeling, including SD obtained by applying 9-fold cross-validation. Both tables show that InselBERT_mammo does not achieve significant improvement compared to the other models.

**Table 3:** Model perplexity scores before and after further pretraining.

Metric	medBERT.de	InselBERT_multi	InselBERT_mammo(3 epochs)	InselBERT_mammo (10 epochs)
Model perplexity	1.21	1.12	1.11	1.09

**Table 4:** Results of fine-tuning for extractive question answering including confidence interval and standard error (confidence level: 0.95-599 resamples).

Metric	medBERT.de, value (95% CI), SE	InselBERT_multi, value (95% CI), SE	InselBERT_mammo (3 epochs), value (95% CI), SE	InselBERT_mammo (10 epochs), value (95% CI), SE
Exact match	79.49 (75.77- 83.3), 1.84	78.02 (74.25- 81.70), 1.91	80.59 (77.33-83.99), 1.69	79.85 (76.33-83.41), 1.85
Averaged *F*_1_-score	90.09 (87.89- 92.46), 1.16	89.78 (87.53- 92.01), 1.13	90.49 (88.36-92.47), 1.06	90.4 (88.28-92.53), 1.11

**Table 5:** Results of fine-tuning for sequence labeling averaged over all classes, based on 9-fold cross-validation.

Metric	medBERT.de, mean (SD)	InselBERT_multi, mean (SD)	InselBERT_mammo (3 epochs), mean (SD)	InselBERT_mammo (10 epochs), mean (SD)
Mean *F*_1_-score	0.81 (0.007)	0.81 (0.007)	0.81 (0.008)	0.81 (0.001)
Mean recall	0.81 (0.008)	0.81 (0.01)	0.82 (0.01)	0.81 (0.007)
Mean precision	0.82 (0.009)	0.81 (0.007)	0.81 (0.01)	0.81 (0.009)
Mean accuracy	0.82 (0.005)	0.82 (0.006)	0.82 (0.006)	0.82 (0.005)

### Qualitative Model Evaluation

The final, dockerized model pipeline had a total size of 3.68 GB and was deployed on an institutional virtual machine without GPU (7.7 Gi RAM and 3.8 Gi Swap). Extracting all facts and entities from a single report took on average less than 20 seconds.

**Figure 3:**
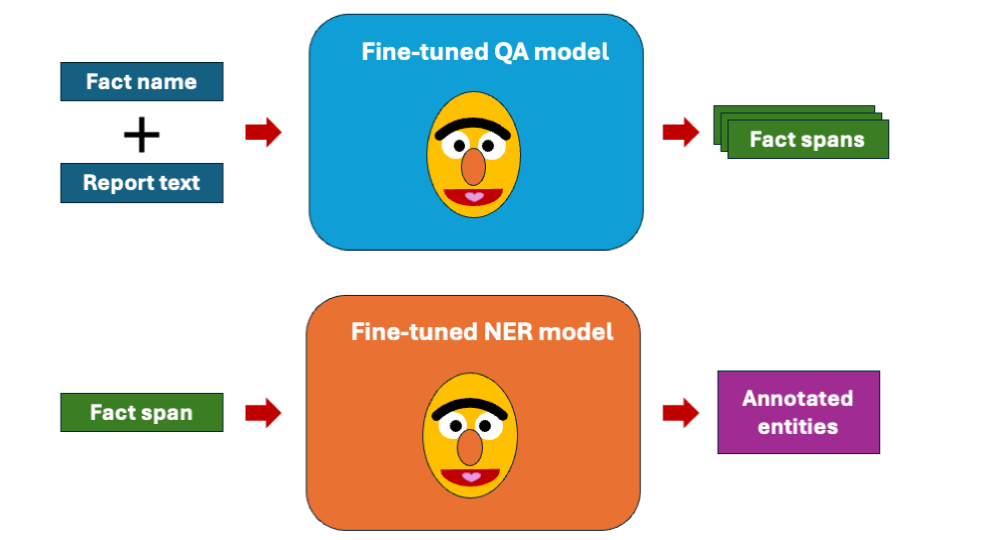
Overview of a single pipeline pass. NER: named entity recognition; QA: question answering.

The pipeline is executed multiple times for each report, once per fact to be extracted, as shown in Figure 3. In each iteration, the fact name and report text are provided as input to the QA model, which then generates a list of fact spans for the given fact name. Next, each extracted fact span is processed by the NER model, which assigns labels to each token according to the predefined fact schema. These annotated entities are used to apply the fact schema template. This is then normalized independently. A detailed overview of this process including examples is given in Multimedia Appendix 6.

Next, this pipeline was used to extract facts and entities from every report in the synthetic dataset. This extraction resulted in a total of 205 extracted facts (14 types), of which 197 (96.1%) were classified correctly. From 497 extracted entities (24 types), 495 (99.6%) were classified correctly. Manual error analysis showed that the misclassification of facts is limited to exactly three text spans corresponding to four fact types (“Mamilla region described,” “Foreign material described,” “Recommendation for further examination,” and “Asymmetry described”) and misclassification of modifiers to the single word “No” and modifier type (“Dignity”). See Table 6 for a detailed overview of all misclassified fact and modifier instances.

Regarding the automated normalization, suitable concepts from two terminologies (SNOMED-CT and Wingert) were retrieved for each extracted entity. Manual verification showed that out of a total of 3582 concepts, 2303 (64.29%) were deemed plausible.

The analysis of the synthetic reports based on the generative approach results in an overall precision of 91.4% and 87.3% for facts and entities, respectively. For implementation details, see Multimedia Appendix 7. Manual error analysis showed that the model tends to hallucinate especially for false positive extractions (ie, when no information is present in the source document). Furthermore, extraction inconsistencies were observed with the model extracting additional information not defined in the fact schema: For example, instead of only extracting the BI-RADS category, the model additionally extracted the size of the lesion as part of the same fact. Analysis of a single report took on average 7 minutes. We have made the results of the analysis available via the Open Science Framework (OSF).

**Table 6:** Misclassified fact and modifier types.

Classified text span	Misclassified fact or modifier types (n)	Correct fact or modifier type
“No retracted areas”	Foreign material described (1), Asymmetry described (3)	Additional result
“No evidence of malignancy”	Foreign material described (1), Recommendation for further examination (2)	Additional result
“Mamilla distance 54mm”	Mamilla region described (1)	Position (mamilla distance)
“No”	Dignity (2)	Negation

## Discussion

### Principal Findings

In this paper, we present a novel, 2-step IE pipeline based on the linguistic concept of frame semantics. First, extractive question answering is used to extract relevant text passages (“facts”) from free-text mammography reports. Next, for each fact, a sequence labeling model identifies a subset of attributes (“modifiers”), specified for each fact type.

Although further pretraining reduces model complexity, it does not have a significant impact on either of the 2 downstream tasks (extractive QA and sequence labeling). Extractive QA reaches an average *F*_1_-score of >90% and sequence labeling reaches a mean *F*_1_-score of >80%.

Using frame semantics in our extraction pipeline helps to organize radiological data in a way that aligns well with clinical practice, by defining frames for common scenarios, the model captures structured relationships between key terms (anchors) and their attributes (modifiers), improving clarity and consistency across reports. This approach makes it easier to add new fact types or modifiers without major adjustments, supporting standardized reporting that can scale effectively to different areas of radiology.

Compared to generative models, our approach has the following advantages, first, model output is directly linked to the original output on a token level, as both fine-tuned models perform sequence labeling on a token basis, effectively prohibiting hallucination. Second, the underlying information model extracts relatively fine-grained information units based on modifiers: This detailed labeling facilitates further processing steps, for example, normalization of terms. Finally, both downstream tasks provide a certainty score for each labeled token, which could be used to dynamically filter out or highlight uncertain predictions.

### Comparison With Previous Work

By using encoder-based model architectures, we ensure that the output of our pipeline is directly linked to the free text in the report provided as input. In the case of model architectures partly or fully based on decoders (also known as generative models), this linkage cannot be directly ensured, but would have to be implemented manually (eg, by prompting the model to provide the text span on which it based its decision on and then verifying if this text span is contained in the original report).

Recent developments in information extraction, such as the GLiNER (Generalist and Lightweight Model for Named Entity Recognition) architecture, demonstrate the increasing versatility of encoder-based models in handling flexible entity definitions and multitask learning [47,48]. GLiNER’s design as a compact encoder-based model allows flexible entity extraction without the need for predefined annotation schemas. While GLiNER excels in scenarios where zero-shot or self-learning approaches are prioritized, evidence for its application in clinical domains currently remains limited to the detection of personally identifiable information and protected health information [49]. This highlights the potential for domain-specific adaptation but lacks the task-specific optimization seen in our pipeline for radiological reports. Performance-wise, GLiNER’s multitask capabilities trade-off precision, as reflected in our observed superior *F*_1_-scores for fact extraction and sequence labeling in a dedicated clinical setting.

Compared to the original IE pipeline proposed by Steinkamp et al, our implementation achieves a similar *F*_1_-score for fact extraction (Steinkamp: 91% over 16 fact types; InselBERT_multi: 90.49% over 14 fact types). According to their description of annotation frequencies, only 2 fact types (“radiologic finding was observed” and “anatomic region has the property”) comprised 73.14% of all fact annotations. A total of 3 fact types comprised less than 20 examples. These were still included in the final evaluation, whereas we decided to exclude all fact instances with less than twenty annotated examples. Unfortunately, performance measures on fact and modifier levels are not reported by the authors. Comparability of both approaches is however limited, as a new set of facts was defined for our study.

While the original paper describing the squad_v2 dataset reported an *F*_1_-score of 66.3% and an Exact Match score of 63.4 %, our approach seems to surpass these values by 24.2% points and 17.2% points, respectively (InselBERT_multi: 90.5%, 80.6%). Interestingly, our pipeline even exceeds human performance with a reported *F*_1_-score of 89.5% by 0.99% points [41]. However, while the squad_v2 dataset contains various free-text questions, our model is restricted to the applied fact schema. Instead of free-text questions, we train the model on fact names. This results in our model only being able to reliably extract the facts it has been trained on. Furthermore, our dataset does not include unanswerable examples. In an unanswerable example, the provided context does not contain the answer to the posed question. Synthetically adding unanswerable examples to our dataset might positively impact model performance.

An important factor to be considered for the real-world application of LLMs is model size, usually determined by the number of parameters: While BERT has 340 million parameters, GPT-4, a recent decoder-only model, has an estimated amount of 1.8 trillion parameters, being approx. 5294 times larger [50]. Although model performance, in general, improves with model size, so does the required amount of training data and hardware resources: MedBERT.de, based on a German BERT variant, was pretrained on a total of approx. 22 GB. GPT-3, the predecessor of GPT-4, however, was pretrained on the CommonCrawl dataset of 45 TB of data [12]. According to Zhang et al [51], pretraining PaLM [a decoder-based model] requires around 2.5×10^24^ floating-point operations and takes 64 days when executed on 6144 Google TPUv4 chips.“ Cloud computing may alleviate these hardware requirements, although especially in health care, its adoption is complicated by existing data protection regulations. Furthermore, as model architectures become larger, the explainability of model outputs, defined as the ”ability to explain or to present in understandable terms to a human“ [52], decreases [20].

Due to the currently high demand for high-performance computing hardware and especially GPUs, smaller models can be trained and inferred on relatively cheap and currently available hardware. This was the approach followed in this work.

Manual evaluation with synthetic data showed that the InselBERT pipeline was able to extract instances from ten out of 14 fact types and 23 out of 24 modifiers types with a precision of 100% each. For the remaining four fact types and the single modifier types that included incorrect prediction, additional annotation might improve model performance. For the case study, prediction probabilities were not considered; increasing the prediction threshold for low-performing types might further increase class-level accuracy. Regarding the experimental normalization of extracted concepts, manual analysis shows that the low overall accuracy of 64.29% is due to a large number of inplausible SNOMED-CT concepts. Performance might be increased by constraining subtype relationships for each entity (eg, or by only using the Wingert terminology). We provide detailed results via the OSF.

### Annotation Process

We put a substantial effort into the generation of the fact schema and the development of annotation guidelines. This was done to allow other research to reuse parts of our proposed fact schema, thereby saving time and resources. In future work, annotation effort might be reduced by applying active learning strategies as described by Jantscher et al [53].

As reported already by other researchers, the annotation process is the limiting factor in approaches like the one presented here. Challenges within the annotation process have already been described by Xia et al [54] and we had similar experiences. Our annotation process presented several challenges, particularly in the communication between informaticians and clinicians. The project encountered issues such as frequent changes in team members and unclear instructions. Future strategies include the generation of silver-standard labels, with clinicians focusing solely on correction. In addition, it is important to note the limited availability of clinicians’ time when designing and implementing annotation projects [55].

From the annotators’ perspective, while numerous components of the radiology reports proved to be formalizable, certain challenges persisted: Structurally, issues such as determining referentiality between phrases and the modification relationships of adjectives presented difficulties. In addition, variability in report-writing conventions across individual clinicians posed considerable obstacles. This variability was particularly problematic given our effort to base annotation guidelines on the BI-RADS criteria for mammographies, resulting in difficulties when annotating reports that did not adhere to these standards.

Nonetheless, the establishment of detailed annotation guidelines enabled thorough annotation of the majority of the clinical findings sections within the radiological reports. We want to emphasize the need to implement an extensive trial phase for clinical annotation projects. This phase should involve (1) the initial development of guidelines by technicians as well as clinicians and (2) subsequent, iterative development and testing of these guidelines. We saw that, after the third iteration, an IAA of >0.7 was effective in resolving ambiguities in the annotation guidelines.

In future studies, it is recommended to separate a guideline development team from an annotation team. In addition, using a larger set of reports (<10) for each iteration is recommended, as increasing the sample size will enhance the generalizability of the guidelines instead of repeating to create specialized rules for a smaller set of reports.

### Limitations

In the following, we point out the limitations of this study to be considered. One major prerequisite of the applied information model is that all information related to a fact is located within one unbroken text span. Therefore, information mentioned outside of facts can currently not be considered. Specifically for the use-case of mammography, we noticed that clinicians report findings for each breast separately. In the future, we want to investigate how to include this external information in the frame semantics approach.

Moreover, due to time constraints during the annotation process, it was decided to only annotate the results sections of the included reports. There might be relevant information in the other sections of the mammography report. To speed up the annotation process, our underlying framework is centered around the idea of reusing existing facts and annotations for other clinical use cases; whether this approach is realistic is subject to an ongoing follow-up project.

Regarding model development, we highlight that our pipeline comprising 2 different models was not trained end-to-end. Instead, we developed and evaluated each of the 2 models separately, before carrying out end-to-end human evaluation using synthetic reports only. Currently, our model is only capable of analyzing German mammography reports. However, using annotation-preserving translation of our training data, our pipeline could be easily adapted to any other language. This task is also known as annotation projection and has been described in the literature [56,57].

Unfortunately, our core dataset cannot be shared due to institutional restrictions: However, it is possible to evaluate our models based on the published synthetic dataset, which should resemble the original reports very well. Furthermore, our pipeline is trained on data obtained by only one institution. We therefore cannot assess the adaptability of our pipeline on external data and generalizability may be limited.

One major limitation regarding the implementation of extractive question answering is the fact that we did not generate unanswerable questions based on our training data. The original dataset published with the metric used in this paper, squad (version 2), contains approximately 30% of unanswerable questions. We hypothesize that by programmatically creating and adding unanswerable entries, performance should further increase. Furthermore, we point out that both downstream tasks are evaluated differently: while extractive QA was developed using a fixed training, testing, and validation split and evaluated using bootstrapping, the sequence labeling model was developed using 9-fold cross-validation and a shared validation set.

### Future Work

We note that there are several open research questions related to our proposed approach that need further investigation and might lead to a performance increase. In this study, only the complete text-span annotated as a fact is used for training the sequence labeling model. We hypothesize that the model could benefit from a bigger context window, including either the whole report or additional leading and trailing tokens surrounding the text span, as implemented by Steinkamp et al [27]. Another strategy to improve sequence labeling performance is concatenating the model input with encoded information on the specific fact type. A similar approach was described by Kuling et al [58] who encoded and concatenated the corresponding report section of each sentence to be labeled.

Another open aspect relates to the sharing of modifiers between fact types as well as experimenting with different modifier aggregation levels: for example, all modifiers describing an anatomical location might be aggregated and shared between all facts. It also remains unclear whether our QA model is capable of inferring new fact types and whether providing a textual description of the fact type instead of its title might improve performance.

Further pretraining of model variants showed little to no effect on downstream task performance. However, as a base model, we applied Medbert.de—a model already pretrained on >4.7 million German medical documents, comprising approx. 3.6 million radiology reports. We hypothesize that our training dataset was simply too small to impact the pretrained model parameters. Increasing further pretraining epochs, changing hyperparameters like learning rate and training a model from scratch solely based on our training dataset are potential strategies for improving model performance that we have not yet investigated.

Finally, we chose an encoder-based approach due to the inherent interpretability of model outcomes as compared to generative models that by design cannot perform token-level predictions. However, recent research shows that including diagnostic reasoning in generative models might improve the assessment of outcomes for clinicians [59]. Due to the high performance and smaller sizes of recent generative models, applying frame semantics to generative models might offer additional advantages.

### Conclusions

In this paper, we demonstrated the feasibility of integrating frame semantics with a BERT-based architecture for extracting information from mammography reports with the purpose of automatic structuring. Our approach allows clinicians to specify the aspects of relevance to be extracted from the reports. In addition, using encoder-based model architectures, the output of our pipeline is directly linked to the free-text in the report provided as input. This supports the explainability of the extraction results, which is crucial in settings where it has to be ensured that the system output is correct. Our evaluation of a generative model-based approach highlights its potential but also its limitations, particularly the occurrence of hallucinations and inconsistencies in fact extraction. These findings reinforce the importance of our structured, token-level approach in ensuring interpretability and precision in medical information extraction. We conclude that even with a small annotated dataset and a relatively small language model, the quality of IE is comparable to human performance. However, challenges remain in handling edge cases, such as semantically overlapping frame definitions and negation detection. The real-world applicability of this approach is still limited by the need for specialized, high-quality annotated data, though model distillation strategies could help mitigating this constraint. In future work, we will validate the results and test the application of the approach to reports from other radiology departments. In addition, the transfer to other examinations and report types will be studied. The suggested approach was designed in a way that the information to be extracted can be defined by clinicians. Together with a dataset annotated according to a new fact schema, the approach could be transferred easily.

## Data Availability

The synthetic dataset, the original annotation guideline in German, all source code, and detailed evaluation results that were generated or analyzed during this study are available in the OSF repository [61] and Zenodo repository [62]. The pretraining and finetuning dataset used during this study is not publicly available due to institutional restrictions.

## Appendix

Multimedia Appendix 1 Example of a synthetic report used for qualitative evaluation.

Multimedia Appendix 2 Example of an annotated report (screenshot from the applied annotation tool Inception). The example comprises five annotated sentences within a single report. Facts are annotated in red, anchor entities in green and modifiers in blue. For overlapping facts, each modifier is additionally linked to all of its corresponding anchor entities as seen in line five. Translation: Dense gland parenchyma. Regular visualization of the cutis, subcutis and nipple region. No suspicious, spiculated focal findings. No suspicious, linear, branched or pleomorphic microcalcifications. No suspicious lymph nodes as far as recorded.

Multimedia Appendix 3 Token characterstics of pre-training data.

Multimedia Appendix 4 Token statistics of fine-tuning data (only mammographies).

Multimedia Appendix 5 Number of annotated fact and modifier instances.

Multimedia Appendix 6 Detailed information flow of the system. The pipeline is run once per fact in the fact schema (green and purple elements denote two separate pipeline runs). A single run (green example) takes the fact name (string) and the report text (string) as an input. The input is tokenized (for simplicity, here each word is converted to a token) and fed into the first component, the fine-tuned question answering model, separated by a [SEP] token (according to the BERT-based architecture). For each input token, the model outputs one start and end logit corresponding to the start and end of the respective fact. For example, for the fact “Cutis described”, the model labels “Regular” as start token and “cutis” as end token. We can then extract the complete fact span within the start and end token. Note that a report text might include multiple instances of the same fact, which are all extracted. Each extracted fact span is then fed into the second component, the fine-tuned sequence-labelling model. For each input token, the model outputs a label corresponding to one of the pre-defined entities according to the BIO (begin, inside, outside) schema, which are then aggregated into entities. For example, for the fact span “Regular display of cutis”, the model labels “Regular display” as state entity and “cutis” as cutis entity. Last, the pre-defined schema for each fact is then filled with the extracted entities.

Multimedia Appendix 7 Implementation details: Generative information extraction.

Multimedia Appendix 8 Prompt used for proofreading and grammar checking of selected paragraphs.

## Abbreviations

ACR: American College of Radiology
BERT: Bidirectional Encoder Representations from Transformers
BI-RADS: Breast Imaging-Reporting and Data System
CAS: common analysis structure
GLiNER: Generalist Model for Named Entity Recognition
IAA: interannotator agreement
IE: information extraction
LLM: large language model
NER: named entity recognition
NLP: natural language processing
OSF: Open Science Framework
QA: question answering
SNOMED-CT: Systematized Nomenclature of Medicine – Clinical Terms
SQuAD_v2: Stanford Question Answering Dataset 2.0
