# Supplementary material for: Enhancing Bidirectional Encoder Representations From Transformers (BERT) With Frame Semantics to Extract Clinically Relevant Information From German Mammography Reports: Algorithm Development and Validation
Source: J Med Internet Res. 2025 Apr 25;27:e68427. doi: 10.2196/68427 (PMC12064967; doi:10.2196/68427)
Supplement: Multimedia Appendix 1 [file jmir_v27i1e68427_app1.docx]

# Example of a synthetic report used for qualitative evaluation

This is a Multimedia Appendix to a full manuscript published in the J Med Internet Res. For full copyright and citation information see [*https://www.jmir.org/2025/1/e68427/*](https://www.jmir.org/2025/1/e68427/)

MAMMOGRAPHIE IN ZWEI EBENEN VOM 11.10.2017.

Fragestellung/ Indikation:

Positive Familienanamnese (Schwester mit 62 Jahren). Tastbefund linke Mamma.

Malignität?

Befund:

Keine Voruntersuchung.

Wenig fibroglanduläres Drüsengewebe beidseits.

Unauffällige Kutis und Subkutis beidseits.

In der linken Mamma umschriebener, runder Herdbefund, 19x12mm gross im unteren inneren Quadranten auf 8 Uhr. Keine Kalzifikation. Mamillendistanz 54mm. Kein weiterer Herdbedund in der linken Mamma. Keine Mikro- oder Makroverkalkungen.

Rechte Mamma ohne Herdbefund, Mikro- oder Makroverkalkung.

Keine suspekten Lymphknoten.

Beurteilung:

Kontrolle des Herdes links empfohlen. Sonst kein Anhalt für ein Malignom.

ACR Typ B beidseits

BIRADS 3 links

BIRADS 1 rechts.
