# Supplementary figures and images for: Enhancing Bidirectional Encoder Representations From Transformers (BERT) With Frame Semantics to Extract Clinically Relevant Information From German Mammography Reports: Algorithm Development and Validation
Source: J Med Internet Res. 2025 Apr 25;27:e68427. doi: 10.2196/68427 (PMC12064967; doi:10.2196/68427)

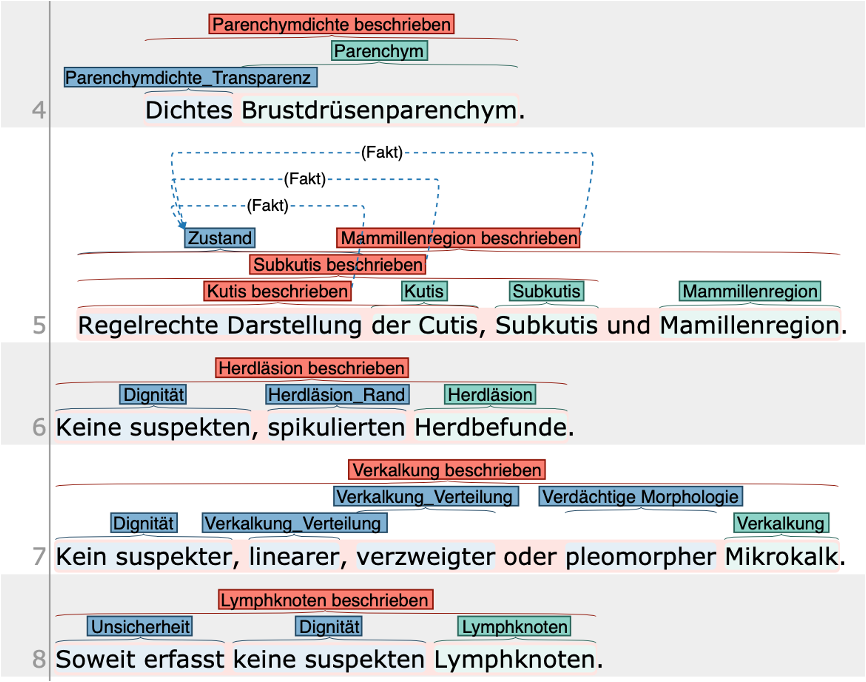

Supplement: Multimedia Appendix 2 [file jmir_v27i1e68427_app2.png]

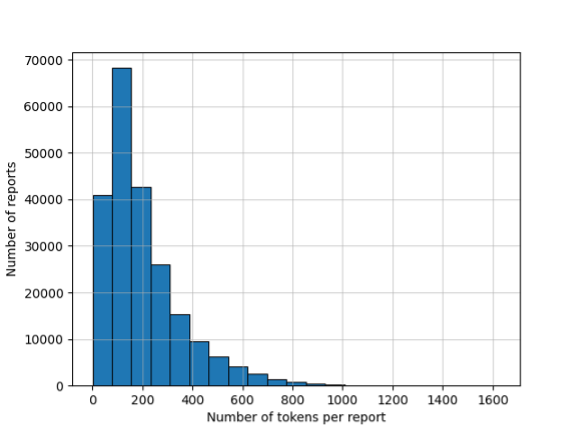

Supplement: Multimedia Appendix 3 [file jmir_v27i1e68427_app3.png]

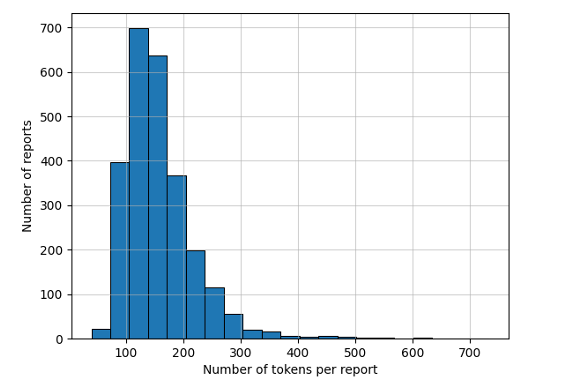

Supplement: Multimedia Appendix 4 [file jmir_v27i1e68427_app4.png]

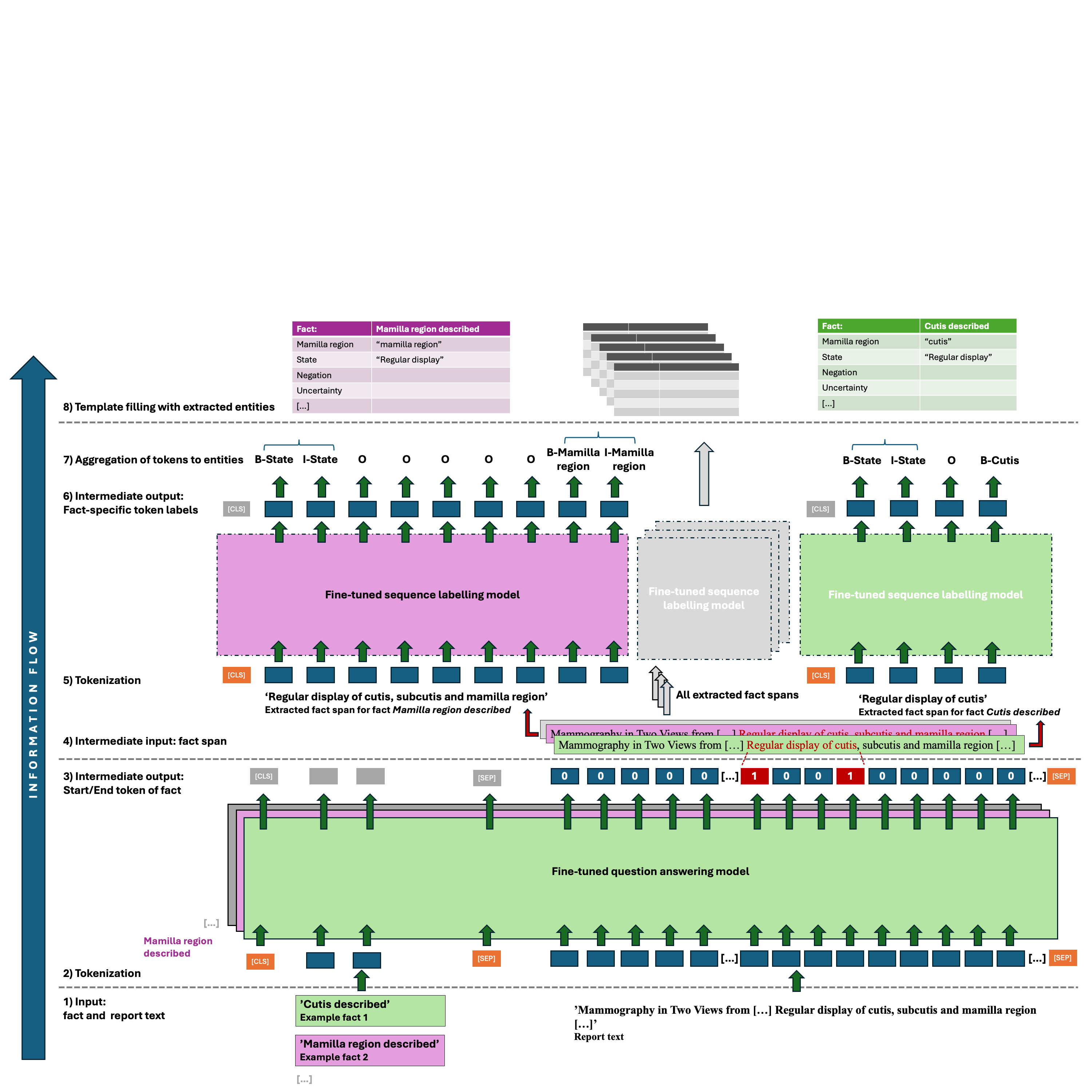

Supplement: Multimedia Appendix 6 [file jmir_v27i1e68427_app6.png]
