# Supplementary material for: Enhancing Bidirectional Encoder Representations From Transformers (BERT) With Frame Semantics to Extract Clinically Relevant Information From German Mammography Reports: Algorithm Development and Validation
Source: J Med Internet Res. 2025 Apr 25;27:e68427. doi: 10.2196/68427 (PMC12064967; doi:10.2196/68427)
Supplement: Multimedia Appendix 5 [file jmir_v27i1e68427_app5.docx]

# Number of annotated fact and modifier instances

This is a Multimedia Appendix to a full manuscript published in the J Med Internet Res. For full copyright and citation information see *<https://www.jmir.org/2025/1/e68427/>*

Table S1. Annotated fact and modifier instances, translated to English. Each fact type implicitly includes an anchor entity not mentioned in this table.

|  | Original name | Translation | No. of annotated instances |
| --- | --- | --- | --- |
|  |  |  |  |
| **Facts** |  |  |  |
|  | Herdläsion beschrieben | Focal lesion described | 479 |
|  | Verkalkung beschrieben | Calcification described | 455 |
|  | BI-RADS Klassifizierung erwähnt | BI-RADS classification mentioned | 346 |
|  | Parenchymdichte beschrieben | Parenchyma density described | 280 |
|  | Kutis beschrieben | Cutis described | 252 |
|  | ACR Klassifizierung erwähnt | ACR classification mentioned | 213 |
|  | Subkutis beschrieben | Subcutis described | 186 |
|  | Lymphknoten beschrieben | Lymph nodes described | 160 |
|  | Mammillenregion beschrieben | Mamilla region described | 110 |
|  | Empfehlung für weitere Untersuchung | Recommendation for further examination | 100 |
|  | Architekturstörung beschrieben | Architectural distortion described | 87 |
|  | Fremdmaterial beschrieben | Foreign material described | 41 |
|  | Asymmetrie beschrieben | Asymmetry described | 34 |
|  | Zusätzlicher Befund | Additional result | 31 |
|  | Vergleich mit Voruntersuchung erwähnt | Comparison with previous examination mentioned | 10 |
|  | Zuweisungsinformation erwähnt | Referral information mentioned | 10 |
|  | Intramammäre Lymphknoten beschrieben | Intramammary lymph nodes described | 8 |
|  | Bildgebendes Verfahren durchgeführt | Imaging procedure conducted | 7 |
|  | Untersuchung hat Limitierung | Examination has limitation | 5 |
|  | Klinischer Befund / Anamnese / Diagnose | Clinical interpretation / medical history/ diagnosis | 2 |
| **Modifiers** |  |  |  |
|  | Lateralität | Laterality | 1576 |
|  | Zustand | State | 661 |
|  | Lokalisierung | Localization | 445 |
|  | Negation | Negation | 373 |
|  | Dynamik | Dynamics | 270 |
|  | Grösse | Size | 224 |
|  | Verkalkung_Verteilung | Calcification (distribution) | 220 |
|  | Dignität | Dignity | 203 |
|  | Position_Uhrzeit | Position (on clock) | 196 |
|  | Parenchymdichte_Transparenz | Parenchyma density (transparency) | 180 |
|  | Position_Mammillenabstand | Position (mamilla distance) | 173 |
|  | Herdläsion_Rand | Focal lesion (rim) | 158 |
|  | Unsicherheit | Uncertainty | 153 |
|  | Position_Quadrant | Position (quadrant) | 133 |
|  | Projektionsebene | Projection plane | 128 |
|  | Verkalkung_Dignität | Calcification (dignity) | 121 |
|  | Begleitmerkmale | Accompanying features | 94 |
|  | Verdächtige Morphologie | Suspicious morphology | 83 |
|  | Anzahl | Count | 80 |
|  | Verdachtsdiagnose | Suspected diagnosis | 63 |
|  | Parenchymdichte_Form | Parenchyma density (form) | 54 |
|  | Diagnoseverfahren | Diagnostic procedure | 48 |
|  | Herdläsion_Form | Focal lesion (form) | 44 |
|  | Zeit / Datum | Time / date | 31 |
|  | Herdläsion_Dichte | Focal lesion (density) | 26 |
|  | Typisch benigne Veränderung | Typically benign mutation | 23 |
|  | Parenchymdichte_Rand | Parenchyma density (rim) | 19 |
|  | Untersuchung | Examination | 19 |
|  | Parenchymdichte_Dignität | Parenchyma density (dignity) | 13 |
|  | Indikation | Indication | 12 |
|  | Verkalkung_Rand | Calcification (rim) | 10 |
|  | Status nach | Status after | 9 |
|  | Zeit / Bedingung | Time / condition | 5 |
|  | Asymmetrie_global | Asymmetry (global) | 3 |
|  | Position_Clip | Position (clip) | 3 |
|  | Asymmetrie_Rand | Asymmetry (rim) | 3 |
|  | Asymmetrie_fokal | Asymmetry (focal) | 2 |
|  | Rand | Rim | 2 |
|  | Abteilung / Arzt | Department / doctor | 1 |
|  | Asymmetrie_progredient | Asymmetry (progressive) | 1 |
