# Supplementary material for: Enhancing Bidirectional Encoder Representations From Transformers (BERT) With Frame Semantics to Extract Clinically Relevant Information From German Mammography Reports: Algorithm Development and Validation
Source: J Med Internet Res. 2025 Apr 25;27:e68427. doi: 10.2196/68427 (PMC12064967; doi:10.2196/68427)
Supplement: Multimedia Appendix 7 [file jmir_v27i1e68427_app7.docx]

# Implementation details: Generative information extraction

This is a Multimedia Appendix to a full manuscript published in the J Med Internet Res. For full copyright and citation information see *<https://www.jmir.org/2025/1/e68427/>*

For information extraction using a generative model, we followed current best-practices in prompt development: As shown beneficial by Van Veen et al. (<https://doi.org/10.1038/s41591-024-02855-5> ), we implemented a few-shot prompt design, augmented by the enforcement of structured output. One prompt is sent per fact class per document, resulting in a total of 1*14*21 = 294 requests. We make the source code available via Zenodo. See Textboxes S1, S2, and S3 for the design of the applied prompt template.

For the few-shot approach, we obtained three random examples from the annotated fine-tuning corpus and integrate them into the system prompt template, see Textboxes 1 and 2. As we cannot share the fine-tuning corpus, we replaces these examples for data sharing with <REMOVED>.

Textbox S1. Prompt template (translated from German).

| You are an experienced radiologist.  Your task is to extract structured information from a free-text radiology report.  This structured information is organized as facts, which represent continuous sections of text.  You will receive a template with the name of a specific fact class and the attributes that need to be filled out.  Return only the specified instances of the fact class. There may be multiple facts in the same document.  Fill in the predefined attributes within a fact section with the exact same text from the report.  If attributes are not present in the text, leave the attribute blank.  Return a JSON object according to the provided template.  Here are a few examples:  {EXAMPLES}  End of examples.  This is the free-text radiology report to be structured:  {DOCUMENT}  This is the fact class whose instances are to be extracted:  {FACT_CLASS}  This is the report template to be filled out:  {REPORT_TEMPLATE}  Think step by step.  RESULT: |
| --- |

Textbox S2. Few-shot template (translated from German). This few-shot template is populated with a report text, the name of the fact class, the empty report template to be populated and the populated report template. Three instances of this few-shot template are created automatically and inserted into the {EXAMPLES} placeholder of the prompt template, see Textbox S1.

| Example:  This is the free-text radiology report to be structured:  {REPORT_TEXT}  This is the fact class whose instances are to be extracted:  {FACT_CLASS}  This is the report template to be filled out:  {TEMPLATE}  RESULT:  {EXAMPLE} |
| --- |

Textbox S3. Empty report template for the fact class “Parenchyma density described” (translated from German).

| {  "fact_class": "Parenchyma density described",  "extracted_fact_instances": [  {  "fact_text": "",  "entities": [  {  "Parenchyma": “"  },  {  "Parenchyma_density_Edge": ""  },  {  "Parenchyma_density_Transparency": “"  },  {  "Parenchyma_density_Shape": ""  },  {  "Parenchyma_density_Dignity": ""  },  {  "Projection_plane": ""  },  {  "Suspected_diagnosis": ""  },  {  "Position_Time": ""  },  {  "Position_Nipple_distance": ""  },  {  "Position_Quadrant": ""  },  {  "Position_Clip": ""  },  {  "Negation": ""  },  {  "Uncertainty": ""  },  {  "Time / Date": ""  },  {  "Laterality": ""  },  {  "Localization": ""  },  {  "Dynamics": ""  },  {  "Condition": ""  },  {  "Size": ""  }  ]  }  ]  } |
| --- |
