# Supplementary material for: Enhancing Bidirectional Encoder Representations From Transformers (BERT) With Frame Semantics to Extract Clinically Relevant Information From German Mammography Reports: Algorithm Development and Validation
Source: J Med Internet Res. 2025 Apr 25;27:e68427. doi: 10.2196/68427 (PMC12064967; doi:10.2196/68427)
Supplement: Multimedia Appendix 8 [file jmir_v27i1e68427_app8.docx]

**Prompt used for proofreading and grammar checking of selected paragraphs**

This is a Multimedia Appendix to a full manuscript published in the J Med Internet Res. For full copyright and citation information see [*https://www.jmir.org/2025/1/e68427/*](https://www.jmir.org/2025/1/e68427/)

*You are an experienced researcher in the domain of medical informatics with a focus on information extraction from radiology reports. Please proofread the following paragraph and point out any spelling or grammatical errors.*

*Here is the paragraph:*

*<Paragraph>*
